# In vitro generation of cytotoxic and regulatory T cells by fusions of human dendritic cells and hepatocellular carcinoma cells

**DOI:** 10.1186/1479-5876-6-51

**Published:** 2008-09-15

**Authors:** Shigeo Koido, Sadamu Homma, Eiichi Hara, Makoto Mitsunaga, Yoshihisa Namiki, Akitaka Takahara, Eijiro Nagasaki, Hideo Komita, Yukiko Sagawa, Toshifumi Ohkusa, Kiyotaka Fujise, Jianlin Gong, Hisao Tajiri

**Affiliations:** 1 Division of Gastroenterology and Hepatology, Department of Internal Medicine, The Jikei University School of Medicine, Tokyo, Japan; 2 Institute of Clinical Medicine and Research, The Jikei University School of Medicine, Tokyo, Japan; 3 Department of Oncology, Institute of DNA Medicine, The Jikei University School of Medicine, Tokyo, Japan; 4 Clinical Data Bank, Institute of DNA Medicine, The Jikei University School of Medicine, Tokyo, Japan; 5 Research Institute for Clinical Oncology, Saitama Cancer Center, Saitama, Japan; 6 Department of Medicine, Boston University School of Medicine, Boston, MA, USA

## Abstract

**Background:**

Human hepatocellular carcinoma (HCC) cells express WT1 and/or carcinoembryonic antigen (CEA) as potential targets for the induction of antitumor immunity. In this study, generation of cytotoxic T lymphocytes (CTL) and regulatory T cells (Treg) by fusions of dendritic cells (DCs) and HCC cells was examined.

**Methods:**

HCC cells were fused to DCs either from healthy donors or the HCC patient and investigated whether supernatants derived from the HCC cell culture (HCCsp) influenced on the function of DCs/HCC fusion cells (FCs) and generation of CTL and Treg.

**Results:**

FCs coexpressed the HCC cells-derived WT1 and CEA antigens and DCs-derived MHC class II and costimulatory molecules. In addition, FCs were effective in activating CD4^+ ^and CD8^+ ^T cells able to produce IFN-γ and inducing cytolysis of autologous tumor or semiallogeneic targets by a MHC class I-restricted mechanism. However, HCCsp induced functional impairment of DCs as demonstrated by the down-regulation of MHC class I and II, CD80, CD86, and CD83 molecules. Moreover, the HCCsp-exposed DCs failed to undergo full maturation upon stimulation with the Toll-like receptor 4 agonist penicillin-inactivated *Streptococcus pyogenes*. Interestingly, fusions of immature DCs generated in the presence of HCCsp and allogeneic HCC cells promoted the generation of CD4^+ ^CD25^high ^Foxp3^+ ^Treg and inhibited CTL induction in the presence of HCCsp. Importantly, up-regulation of MHC class II, CD80, and CD83 on DCs was observed in the patient with advanced HCC after vaccination with autologous FCs. In addition, the FCs induced WT1- and CEA-specific CTL that were able to produce high levels of IFN-γ.

**Conclusion:**

The current study is one of the first demonstrating the induction of antigen-specific CTL and the generation of Treg by fusions of DCs and HCC cells. The local tumor-related factors may favor the generation of Treg through the inhibition of DCs maturation; however, fusion cell vaccination results in recovery of the DCs function and induction of antigen-specific CTL responses in vitro. The present study may shed new light about the mechanisms responsible for the generation of CTL and Treg by FCs.

## Background

Hepatocellular carcinoma (HCC) is one of the most common cancers with a rapidly progressive clinical course and a poor prognosis [1,2]. Although several treatments such as tumor resection, liver transplantation, transcatheter arterial chemoembolization (TAE), and local radiofrequency ablation (RFA) are now used to treat HCC, there is no overall long-term survival benefit so far [3,4]. Therefore, therapy to prevent the recurrence of HCC is essential. In this context, immunotherapy represents a potential approach for eradicating the residual tumors in patients with HCC. In support of the immunotherapy approach is the finding that HCC cells overexpress the α-fetoprotein (AFP), NY-ESO-1, carcinoembryonic antigen (CEA), WT1, and glypican-3 as potential targets for the induction of antigen-specific cytotoxic T lymphocytes (CTL) responses [5-9]. It has been reported that vaccination of HCC patients is effective for preventing postoperative recurrence of HCC [10-12].

Because dendritic cells (DCs) are the most potent antigen presenting cells (APCs) and attractive vectors for cancer immunotherapy, the uses of DCs as a booster of antitumor responses have been considered a promising strategy for cancer vaccine. Different strategies to introduce tumor-associated antigens (TAAs) into DCs have been applied to elicit and boost the antitumor immune responses [13-18]. Although clinical trials have demonstrated immunological and clinical responses after vaccination with DCs pulsed with tumor specific peptides, a major drawback of this strategy comes from a limited number of known tumor peptides available in many HLA contexts and the potential evasion of immunological targeting through their antigens down-regulation. To solve this problem, an alternative approach has been developed by fusing DCs with tumor cells [19]. In this approach, a broad spectrum of TAAs, including those known and unidentified, can be fully presented by MHC class I and II molecules in the context of costimulatory molecules [19-25]. Although vaccination with FCs was associated with immunological responses, the clinical responses from early clinical trails in patients with melanoma, glioma, gastric, breast, and renal cancer was muted [20-33].

CTL play a central role in induction of antitumor immunity. Indeed, a high frequency of CD8^+ ^CTL infiltrating cancer tissue can be a favorable prognostic indicator in HCC [34]. However, the progression of tumors despite the presence of infiltrating CD8^+ ^CTL suggests that immunological tolerance is induced, at least in part, by tumors. Recent studies have suggested that increased CD4^+^α chain of IL-2R (CD25)^+ ^forkhead box P3 (Foxp3)^+ ^regulatory T cells (Treg) impair the effector function of CD8^+ ^CTL and are associated with HCC invasiveness [35]. The tumor microenvironment may play an important role in the recurrence and survival of HCC. Therefore, the mechanisms by which Treg arise in vivo and exert their immunoregulatory effects remain to be defined and are the subject of intensive investigation.

In the present study, we first show that coculture of T cells from healthy donors with the fusion cells (FCs) created by allogeneic HCC cells and immature DCs from the donors (DCs/allo-HCC) results in activation of both CD4^+ ^and CD8^+ ^T cells, as demonstrated by high levels of IFN-γ production and lysis of the CEA- and/or WT1-positive targets restricted in HLA-A2 and/or HLA-A24. Interestingly, fusions of immature DCs generated in the presence of HCC cell culture supernatants (HCCsp) and allogeneic HCC (DCs/allo-HCC/sp) induce dysfunction of the fused cells and promote the generation of CD4^+ ^CD25^high ^Foxp3^+ ^Treg and impair the induction of antigen-specific CTL in the presence of the supernatants. Finally, we show that vaccination of the HCC patient with autologous FCs (DCs/auto-HCC) is associated with enhanced immunological responses, as demonstrated by: 1) augmented DCs function; 2) improved production of IFN-γ in both CD4^+ ^and CD8^+ ^T cells and T-cell proliferation; 3) enhanced induction of CEA and/or WT1-specific CTL responses; and 4) augmented CTL activity against autologous HCC cells in vitro assay.

## Methods

### Cell lines

K562 cells (American Type Culture Collection) were maintained in DMEM medium. Colorectal carcinoma cell lines (COLP-2 and COLM-6) were maintained in TIL Media I medium (IBL, Takasaki, Japan) [33]. All media were supplemented with 10% heat-inactivated FCS, 2 mM L-glutamine, 100 units/ml penicillin, and 0.1 mg/ml streptomycin.

### Generation of monocyte-derived DCs

Monocyte-derived DCs from healthy donors (obtained with following informed consent and approved by our institutional review board) were generated. In brief, peripheral blood mononuclear cells (PBMCs) were prepared from whole blood by Ficoll density-gradient centrifugation. The PBMCs were suspended in tissue culture flask in RPMI 1640 supplemented with 1% heat inactivated autologous serum for 60 minutes at 37°C to allow for adherence. The nonadherent cells were removed and the adherent cells were cultured overnight. To generate immature DCs (DCs), the nonadherent and loosely adherent cells were collected on the next day and placed in RPMI 1640 medium containing 1% heat-inactivated autologous serum, 1000 U/ml recombinant human GM-CSF (Becton Dickinson, Bedford, MA, USA), and 500 U/ml recombinant human IL-4 (Becton Dickinson) for 6 days. To assess the effects of HCCsp on DCs generation, we have created four types of DC preparation: 1) DCs; 2) DCs generated in the presence of HCCsp during the entire culture period (DCs/sp); 3) DCs exposed to 0.1 KE/ml (0.1 KE equals of 0.01 mg of dried streptococci) penicillin-inactivated *Streptococcus pyogenes *(OK-432) (Chugai Pharmaceutical) for 3 days (OK-DCs) as described previously [25]; 4) OK-DCs generated in the presence of HCCsp during the entire culture period (OK-DCs/sp). Four types of DC were generated in the presence of equal amounts of GM-CSF and IL-4 during the entire culture.

To generate monocyte-derived DCs for vaccination, PBMCs derived from the HCC patient were freshly isolated (obtained with following informed consent and approved by our institutional review board). Autologous DCs were generated in RPMI 1640 medium containing 1% heat-inactivated autologous serum, 1000 U/ml recombinant human GM-CSF, 500 U/ml recombinant human IL-4, and 10 ng/ml recombinant TNF-α (Becton Dickinson) [30]. On day 6 of culture, DCs harvested from the nonadherent and loosely adherent cells were used for fusion. The firmly adherent monocytes were harvested and used as an autologous target for the CTL assays.

### HCC cell culture and supernatants

The HCC patient was a 54-year-old man with chronic active hepatitis based on carrier state of hepatitis B virus (HBsAg+, HBsAb-, HBeAg-, HBeAb+, HBcAb+, and HCVAb-). Hepatic resection was carried and histological examination revealed moderately differentiated HCC. Specimen from resected HCC (obtained with following informed consent and approved by our institutional review board obtained) was isolated and maintained in TIL Media I medium with 10% heat-inactivated FCS, 2 mM L-glutamine, 100 U/ml penicillin, and 0.1 mg/ml streptomycin. The HCC cells were used for fusion cell preparations created with DCs either from healthy donors or the HCC patient. The HCC cell culture supernatants (HCCsp) were collected at 70–80% confluence. After centrifugation at 1200 rpm for 10 min, HCCsp were passed through a 0.45 um filter. We used HCCsp to investigate whether HCCsp influence the differentiation of FCs and their ability to generate CTL or Treg. Moreover, vaccination with fusions of the patient-derived DCs and autologous HCC cells was started after 5 month of operation (with following informed consent and approval of clinical protocols by our Institutional Review Board (No. 10–33 (2678)).

### Fusions of DCs and allogeneic HCC cells

DCs from healthy donors were harvested and mixed with the HCC cells at a ratio of 10:1. The mixed cell pellets were gently resuspended in PEG (molecular weight = 1,450)/DMSO solution (Sigma-Aldrich St. Louis, MO) at room temperature for 3 to 5 minutes. Subsequently, the PEG solution was diluted by slow addition of serum-free RPMI 1640 medium. The cell pellets were resuspended in prewarmed RPMI 1640 medium supplemented with 10% heat-inactivated autologous serum containing GM-CSF and IL-4 for 3 days [27,33]. To examine the effects of HCCsp on fusion cell generation, fusion cell preparations were exposed to HCCsp during the entire culture period in the presence of equal amounts of GM-CSF and IL-4. We have created four types of FC preparation: 1) DCs fused with allogeneic HCC cells in the absence of HCCsp during the entire culture (DCs/allo-HCC); 2) DCs/sp fused with allogeneic HCC cells in the presence of HCCsp during the entire culture (DCs/allo-HCC/sp); 3) OK-DCs fused with allogeneic HCC cells in the absence of HCCsp during the entire culture (OK-DCs/allo-HCC); and 4) OK-DCs/sp fused with allogeneic HCC cells in the presence of HCCsp during the entire culture (OK-DCs/allo-HCC/sp).

### Vaccination of the HCC patient with autologous FCs

DCs from the HCC patient were freshly fused with autologous HCC cells for each vaccination [27,33]. Autologous FCs were irradiated, suspended in 0.3 ml normal saline, and underwent up to nine times vaccinations via SC injection in the left inguinal area at 2-week intervals [29,30]. The number of DCs used for the generation of fusions was 1–2 × 10^6 ^in each vaccination. The patient was monitored and underwent serial measurements of antinuclear antibodies to assess for evidence of autoimmunity.

### Phenotype analysis

Cells were incubated with FITC- conjugated Abs against-CEA (B1.1), MUC1 (HMPV), MHC class I (W6/32), MHC class II (HLA-DR), B7-1 (CD80), B7-2 (CD86) (BD Pharmingen), HLA-A2, or HLA-A24 (One Lambda). After washing with cold PBS, cells were fixed with 2% paraformaldehyde. For WT1 staining, cells were permeabilized (Cytofix/Cytoperm) and stained with FITC-conjugated anti-WT1 polyclonal Ab (C-19, Santa Cruz, CA). For analysis of dual expression, cells were stained with PE- conjugated anti-HLA-DR, washed, permeabilized, and incubated with FITC- conjugated anti-WT1. Cells were washed, fixed, and analyzed by FACScan (Becton Dickinson, Mountain View, CA) with FlowJo analysis software.

### T-cell proliferation assay

Nonadherent PBMCs from healthy donors were cultured with unirradiated DCs/allo-HCC at a ratio of 10:1 for 3 days in the absence of HCCsp in complete RPMI 1640 medium supplemented with 10% heat-inactivated FCS, 100 units/ml penicillin, and 0.1 mg/ml streptomycin. DCs alone, the HCC cells alone, an unfused mixture of both DCs and the HCC cells were used as controls. T cells were purified with nylon wool and cultured for an additional 4 days in the presence of recombinant human IL-2 (20 units/ml, Shionogi, Osaka, Japan). To assess the effects of HCCsp on T-cell stimulation, nonadherent PBMCs were stimulated by unirradiated DCs/allo-HCC/sp in the presence of HCCsp for 3 days. On day 4 of culture, T cells were purified with nylon wool and cultured for an additional 4 days in the presence of recombinant human IL-2 (20 units/ml). In this case, T cells were cultured in the presence of HCCsp at the initiation and subsequently during the entire culture. Moreover, to assess the ability of autologous FCs vaccination to stimulate T cells, PBMCs (before vaccination and one month after the ninth vaccination) were isolated and cryopreserved in liquid nitrogen in the presence of 10% DMSO/90% autologous serum. Autologous PBMCs were thawed, washed, and plated at 1 × 10^6 ^cells/well in a 24-well plate. Next day, nonadherent PBMCs were cocultured with DCs, the HCC cells, an unfused mixture of both DCs and the HCC cells, or unirradiated DCs/auto-HCC at a ratio of 10:1 in the absence of HCCsp for 3 days. On day 4 of culture, T cells were purified with nylon wool and cultured for an additional 4 days in the presence of recombinant human IL-2 (20 units/ml). On day 8 of culture, T cells were cultured in 96-well U-bottomed culture plates at indicated numbers/well. Dye solution was added to each well and incubated for 4 hr according to the protocol of Cell Titer 96 Non-radioactive Cell Proliferation Assay Kit (Promega, Madison, WI). For measurement, we used the Microplate Imaging System (Bio-Rad, Hercules, CA) at an OD of 550 nm.

### CD4^+ ^CD25^+ ^Foxp3^+ ^staining

For analysis of CD4^+ ^CD25^+ ^Foxp3^+ ^T cells, Foxp3 Staining Kit was used according to manufacture's instructions (BD Pharmingen). Briefly, T cells were incubated with FITC- conjugated anti-CD25 mAb (2A3) and PE-Cy-5-conjugated anti-CD4 mAb (RPA-T4). After wash, intracellular staining was performed with PE-conjugated anti-Foxp3 mAb (259D/C7), washed, and analyzed by FACScan (Becton Dickinson, Mountain View, CA) with FlowJo analysis software.

### IFN-γ and IL-10 production in CD4^+ ^and CD8^+ ^T cells

For analysis of IFN-γ or IL-10 production, each cytokine secretion assay kit was used according to manufacture's instructions (Miltenyi Biotec, Auburn, CA). Briefly, T cells were washed with cold PBS and incubated with cytokine catching reagent for 5 minutes at 4°C. After incubation, 10 ml of prewarmed complete medium was added with shaking and cultured for 45 minutes at 37°C. After incubation, cells were labeled with PE-conjugated cytokine detection antibody for 20 minutes on ice and further stained with FITC-conjugated anti-CD4 or CD8 mAb (Miltenyi Biotec) for 20 min on ice. IFN-γ or IL-10 labeled T cells were washed, fixed and analyzed by two-color FACScan analysis using CellQuest analysis software (BD Biosciences). The reactivity of CD4^+ ^or CD8^+ ^T cells to produce IFN-γ is shown as the percentage of the total population of CD4^+ ^or CD8^+ ^T cells that were positive for IFN-γ.

### Pentameric assays

Pentameric assays of soluble class I MHC-peptide complexes were used to detect antigen-specific CTL activity induced by vaccination with autologous FCs. Complexes of PE-conjugated HLA-A2-WT1 pentamer (126–134, RMFPNAPYL), HLA-A2-CEA pentamer (571–579, YLSGANLNL), or irrelevant pentamer were used (PROIMMUNE Oxford, UK). The pentameric staining was performed according to the manufacturer's instructions. Briefly, the stimulated T cells were incubated with PE-conjugated pentamer for 10–15 minutes at room temperature. After washing with PBS, FITC-conjugated anti-CD8 mAb was incubated for 20–30 minutes at 4°C. Cells were washed, fixed and analyzed by FACScan using CellQuest analysis software (BD Biosciences). The reactivity of CD8^+ ^T cells to WT1 or CEA or both are shown as the percentage of the total population of CD8^+ ^T cells that were double positive (CD8^+^pentamer^+^).

### Cytotoxicity assays

The cytotoxicity assays were performed by flow cytometry CTL assay that was predicted on measurement of CTL-induced caspase-3 activation in the target cells through detection of the specific cleavage of fluorogenic caspase-3 using Active Caspase-3 Apoptosis Kit I (BD Pharmingen) [36,37]. The target cells including the HCC cells, allogeneic tumor cell lines, autologous monocytes, and NK-sensitive K562 cells were labeled with the red fluorescence dye PKH-26 (Sigma, St. Louis, MO). After washing with PBS, PKH-26-labeled target cells were cultured with T cells for 2 h at 37°C in 96 well V-bottom plates. In certain experiments, PKH-26 labeled target cells were pre-incubated with anti MHC class I mAb (W6/32; 1:100 dilution), or control IgG for 30 minutes at 37°C before addition of effector cells. Cells were washed, fixed with Cytofix/Cytoperm Solution (BD Pharmingen) and then washed with Perm/Wash Buffer (BD Pharmingen). Cells were incubated with FITC-conjugated anti-human Active Caspase-3 substrate (BD Pharmingen) for 30 minutes at room temperature, followed by 2 washes with Perm/Wash Buffer. The percentage of cytotoxicity (mean ± SD of 3 replicates) was determined by the following calculation: percentage of Caspase-3 staining = [(Caspase-3^+^PKH-26^+ ^cells)/(Caspase-3^+ ^PKH-26^+ ^cells + Caspase-3^-^PKH-26^+ ^cells)] × 100.

### Statistical analysis

The Student *t *test was used to compare various experimental groups. A *p *value <0.05 was considered to be statistically significant.

## Results

### Phenotypic characterization of DCs generated in the presence of HCCsp

**Figure 1:**
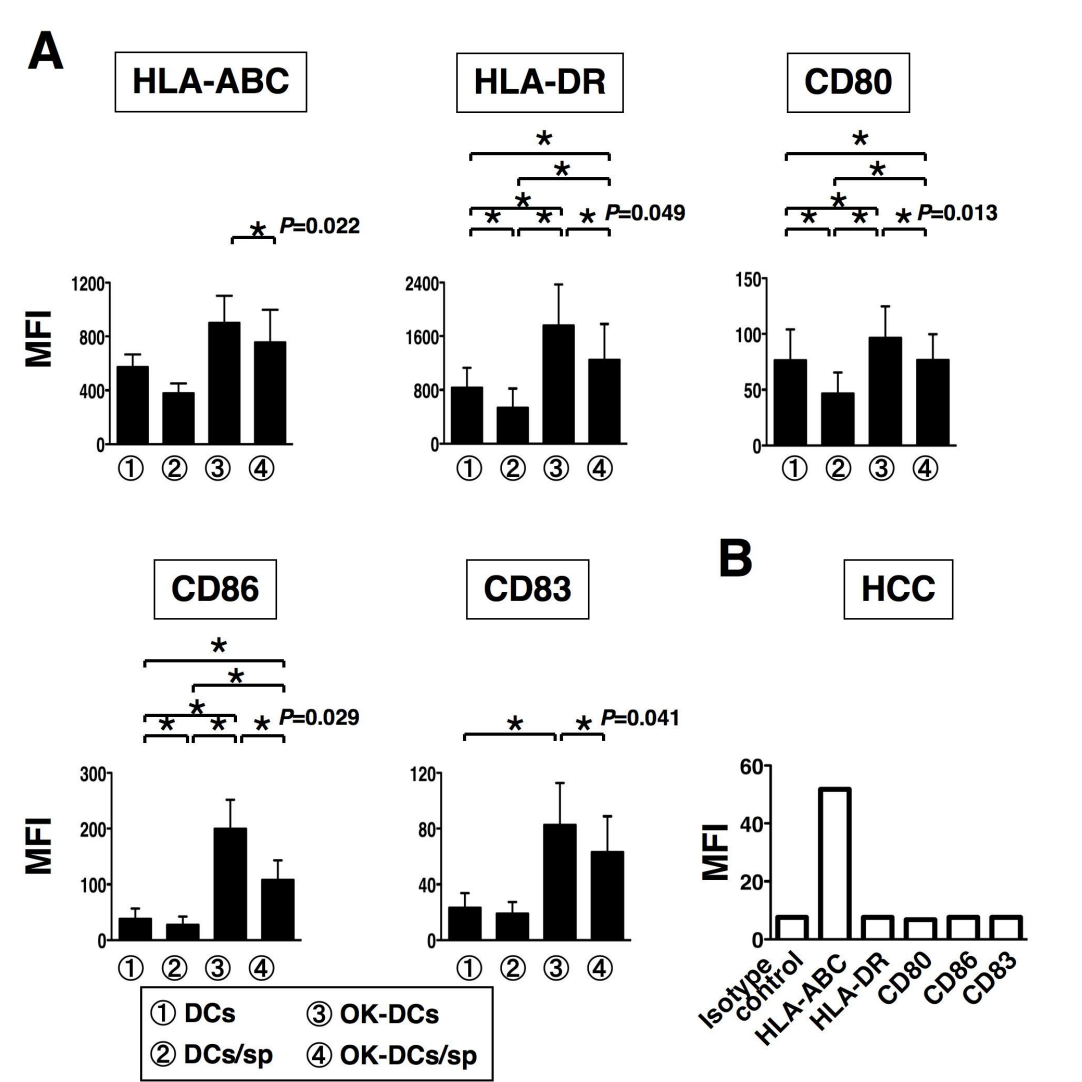
**Inhibition of the differentiation of DCs by HCCsp**. *A*, We have created four types of DC from four healthy donors; 1) DCs; 2) DCs/sp; 3) OK-DCs; and 4) OK-DCs/sp. MFIs of HLA-ABC, HLA-DR, CD80, CD86, and CD83 in four types of DC were analyzed. For each group of DCs, the mean ± SD is shown. *, Significant differences. *P *value (OK-DCs vs OK-DCs/sp) is represented. *B*, MFIs of isotype control, HLA-ABC, HLA-DR, CD80, CD86, and CD83 in the HCC cells were analyzed.

Monocyte-derived DCs from healthy donors were generated in the presence of GM-CSF and IL-4. To assess the effects of HCCsp on DCs generation, we have prepared four types of DC preparation; 1) DCs; 2) DCs/sp; 3) OK-DCs; and 4) OK-DCs/sp. Mean fluorescence intensity (MFI) of HLA-ABC, HLA-DR, CD80, CD86, and CD83 by four types of DC was determined by FACS analysis. The DCs displayed a characteristic phenotype with expression of HLA-ABC, HLA-DR, costimulatory molecules (CD80 and CD86), but low levels of the maturation marker, CD83 (Figure 1A). OK-DCs, as compared with DCs, expressed much higher levels of HLA-DR, CD80, CD86, and CD83 (Figure 1A). Interestingly, DCs generated in the presence of the supernatants (DCs/sp) were associated with down-regulation of antigen presenting molecules, including HLA-DR, CD80, and CD86 (Figure 1A). To evaluate the effects of HCCsp on the activation of DCs, DCs/sp were also subsequently stimulated with the Toll-like receptor (TLR) 4 agonist, OK-432 for 3 days (OK-DCs/sp). OK-DCs/sp, as compared with OK-DCs, exhibited much lower expression of HLA-ABC, HLA-DR, CD80, CD86, and CD83. Therefore, even if DCs/sp were exposed to a stimulus such as OK-432, these DCs could not express full levels of costimulatory molecules and the maturation marker in the presence of HCCsp.

### Effect of HCCsp on the phenotype of fusion cell preparations

**Figure 2:**
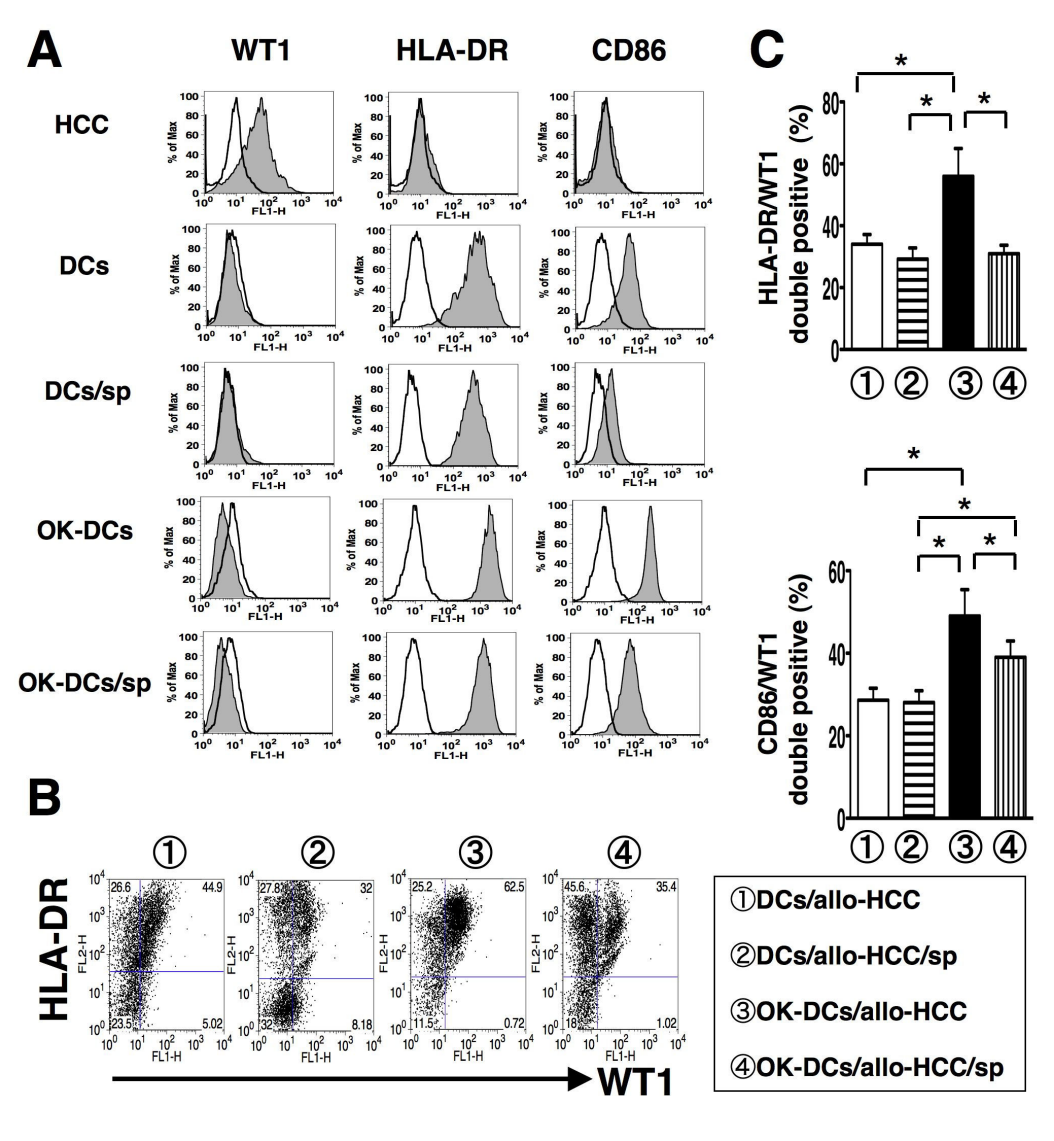
**Phenotypic analysis of DCs/allo-HCC fusion cells created in the presence of HCCsp**. *A*, Four types of DC were analyzed by flow cytometry for expression of the indicated antigens (tinted area) *B*, Four types of FC preparation 1) DCs/allo-HCC; 2) DCs/allo-HCC/sp; 3) OK-DCs/allo-HCC; and 4) OK-DCs/allo-HCC/sp were analyzed by two-color flow cytometry for expression of WT1 and HLA-DR. Numbers represent cells positively staining for the indicated surface markers. *C*, Percentage of cells positive for WT1 and HLA-DR in four types of FC preparation from three healthy donors was analyzed. For each group, the mean ± SD is shown. *, Significant differences.

To assess the effects of HCCsp on DCs/tumor fusion cell generation, we have created four types of FC preparation: 1) DCs/allo-HCC; 2) DCs/allo-HCC/sp; 3) OK-DCs/allo-HCC; and 4) OK-DCs/allo-HCC/sp. DCs displayed a characteristic phenotype with expression of HLA-ABC, HLA-DR, CD80, CD86, and CD83 molecules (Figure 1A and 2A). However, the HCC cells used for fusion expressed high levels of WT1 and HLA-ABC and low levels of CEA but not HLA-DR, CD80, CD86, and CD83 molecules (Figure 1B, 2A, and 5A). Fusions of DCs to the HCC cells coexpressed the HCC cells-derived WT1 antigens and DCs-derived HLA-DR and costimulatory molecules (Figure 2B and 2C). The fusion efficiency was determined by dual expression of tumor marker, WT1, and DC marker, HLA-DR. The cells positive for both WT1 and HLA-DR in OK-DCs/allo-HCC increased when compared with those in DCs/allo-HCC (Figure 2B and 2C). These results support our previous finding that OK-432 promotes fusion efficiency [25]. However, the percentage of double-positive cells (WT1 and HLA-DR/CD86) in OK-DCs/allo-HCC/sp was significantly decreased. These results suggest that soluble factors derived from the HCC cells have detrimental effect on the expression of maturation molecules of DCs/tumor fusion cells.

### Induction of HCC cells-specific CTL by DCs/allo-HCC

**Figure 3:**
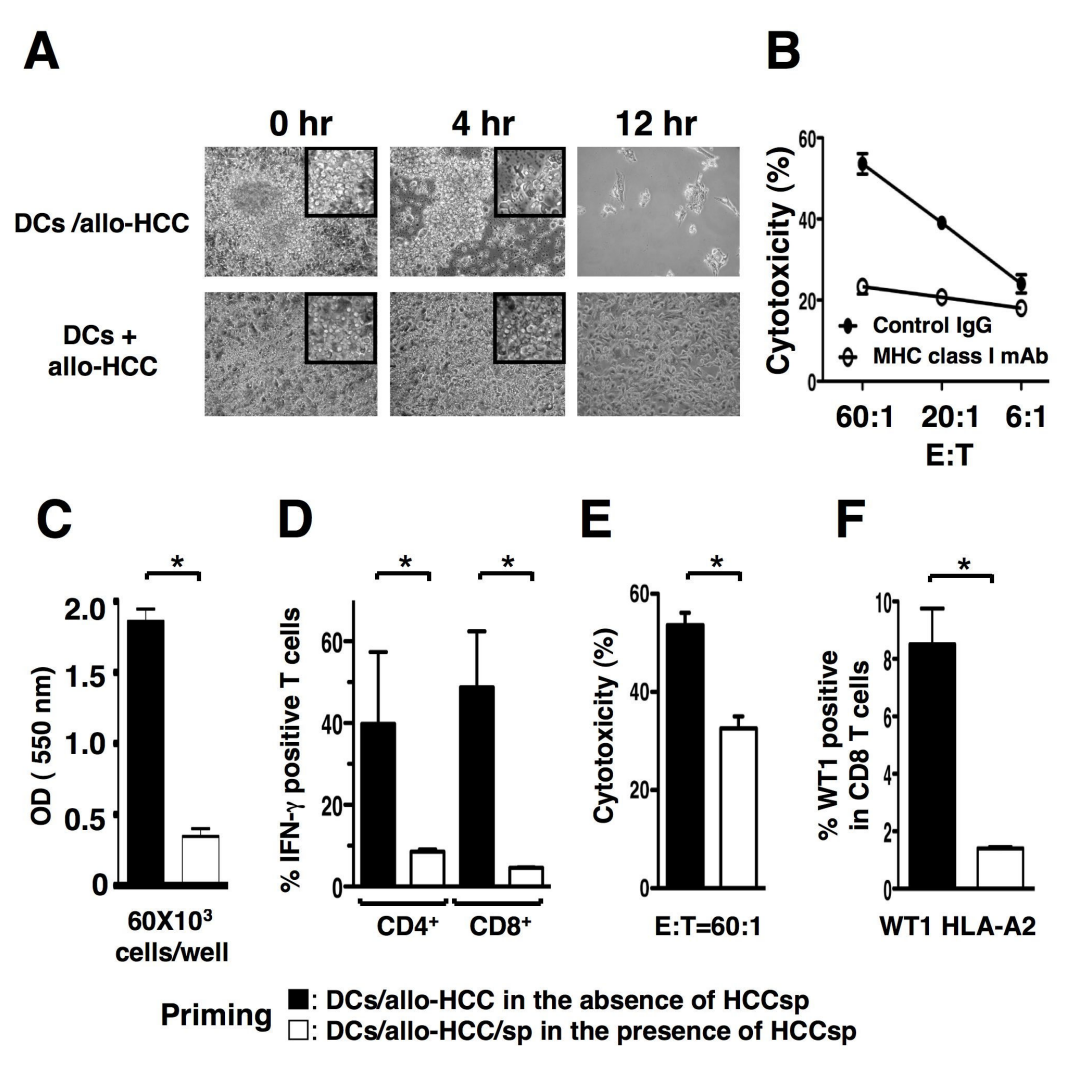
**Induction of HCC cells-specific CTL by DCs/allo-HCC**. *A*, Nonadherent PBMCs from healthy donors stimulated by unirradiated DCs/allo-HCC (upper panel) or DCs mixed with allo-HCC cells (lower panel) were cocultured with the HCC cells at a ratio of 10:1. After 4 hr culture, cells were examined under a microscope. After 12 hr culture, floating cells were harvested and adherent cells were examined (magnification, ×20) (magnification, × 100 in upper corner panel). *B*, T cells stimulated by unirradiated DCs/allo-HCC were cocultured with PKH26-labeled allo-HCC cells at the indicated ratios. Target cells were preincubated with control IgG (solid circles) or anti-MHC class I mAb (W6/32; 1:100 dilution, open circles).*C*, T cells were stimulated with 2 types of fusion cell preparation from three healthy donors: 1) unirradiated DCs/allo-HCC in the absence of HCCsp (■) and 2) unirradiated DCs/allo-HCC/sp in the presence of HCCsp (□). T-cell proliferation assay was performed by Cell Titer 96 Nonradioactive Cell Proliferation Assay Kit according to the protocol. *D*, After stimulation with the 2 types of fusion cell preparation from four healthy donors, percentage of IFN-γ-positive CD4^+ ^or CD8^+ ^T cells was assessed. *E*, After stimulation with the 2 types of fusion cell preparation from three healthy donors, T cells were incubated with PKH-26 labeled allo-HCC cells at a ratio of 60:1. Percentage of cytotoxicity (mean ± SD of 3 replicates) was determined by flow cytometry-CTL assay. *F*, After stimulation with the 2 types of fusion cell preparation, CD8^+ ^T cells (HLA-A2+) from three healthy donors were analyzed by HLA-A2/WT1 pentameric assay. CD8+ T cell reactivity to WT1 was shown as the percentage of double-positive population (CD8+ pentamer+) among all CD8+ T cells. For each group, the mean ± SD of three experiments is shown. *, Significant differences.

To determine whether HCC cells-reactive T cells are induced by fusion cells, T cells from healthy donors were stimulated by fusions of DCs from the same healthy donors (HLA-A2+) and the HCC cells (HLA-A2+, WT1+, and CEA+) (DCs/allo-HCC). Cytotoxicity was assessed with flow cytometry CTL assays that were predicated on measurement of CTL-induced caspase-3 activation in target cells through detection of specific cleavage of fluorogenic caspase-3 [36,37]. The fusion cells could prime naive T cells to differentiate into CTL with lytic activity against the HCC cells (Figure 3A and 3B). After 4 hr coculture of the HCC cells with healthy donor's T cells stimulated by unirradiated DCs/allo-HCC, the majority of the HCC cells were detached (Figure 3A, middle panel). Almost all of the HCC cells were killed after 12 hr incubation (Figure 3A, right panel). The lysis was inhibited by preincubation of target cells with an anti-HLA-ABC mAb, indicating restriction by MHC class I (Figure 3B). By contrast, T cells stimulated by an unfused mixture of both DCs and the HCC cells failed to detach the HCC cells (Figure 3A).

To assess whether the exposure of HCCsp affects the stimulating ability of fusion cells, the HCC cells were fused to DCs/sp from healthy donors in the presence of the supernatants during the entire culture (DCs/allo-HCC/sp). The fusion cells have inferior ability to stimulate the proliferation of T cells (Figure 3C) that expressed lower levels of IFN-γ (Figure 3D) and to induce CTL responses against the HCC cells (Figure 3E), suggesting that the soluble factors in the supernatant inhibit the maturation of fusion cells and have a negative impact in the stimulation of T cells.

To determine the induction of WT1-specitic CD8^+ ^T cells, a pentameric assay of soluble class I MHC-peptide complexes was used to detect the antigen-specific CTL. After stimulation with unirradiated DCs/allo-HCC, 8.5 ± 2.18% of CD8^+ ^T cells were positive for WT1 (Figure 3F). In contrast, the frequency of WT1 pentamer-binding CD8^+ ^T cells among CD8^+ ^T cells decreased to 1.4 ± 0.08% when stimulated by unirradiated DCs/allo-HCC/sp in the presence of HCCsp (Figure 3F). There were no pentamer-positive CD8^+ ^T cells when control epitope pentamer was used or T cells were stimulated by an unfused mixture of DCs and the HCC cells (data not shown). These results suggest that the induction of antigen-specific T cells is affected by HCCsp during T cell-stimulation.

### Generation of CD4^+ ^CD25^high ^Foxp^3+ ^Treg by DCs/allo-HCC/sp

**Figure 4:**
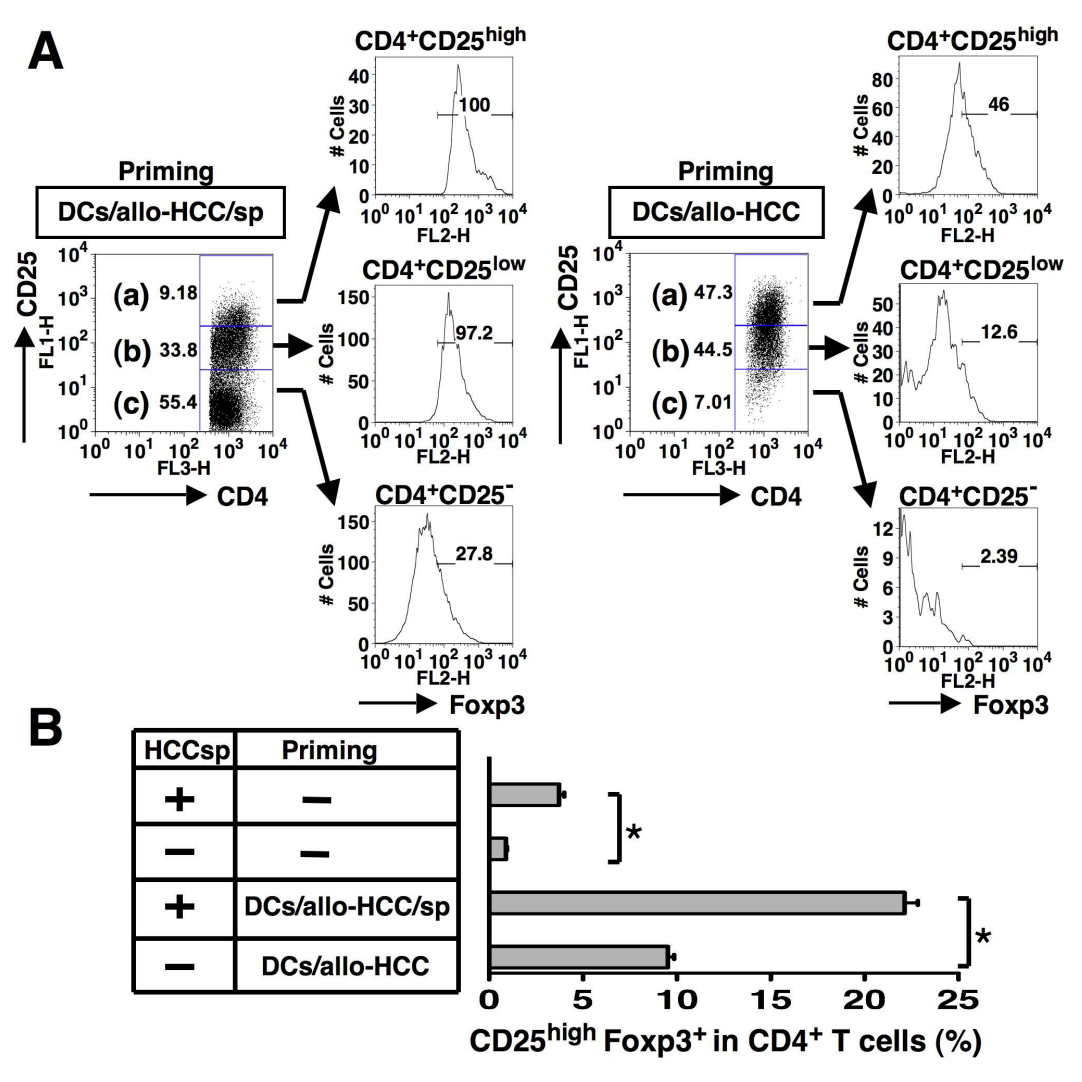
**Generation of CD4^+ ^CD25^+^Foxp3^+ ^Treg in the presence of HCCsp**. *A*, Nonadherent PBMCs were stimulated with unirradiated DCs/allo-HCC in the absence of HCCsp (right panel) or unirradiated DCs/allo-HCC/sp in the presence of HCCsp (left panel). On day 4, T cells were purified, cultured, and analyzed by 3-color flow cytometry for expression of CD4, CD25, and Foxp3. Three different populations; a) CD4^+^CD25^high ^T cells; b) CD4^+^CD25^low ^T cells; c) CD4^+^CD25^- ^T cells were gated to analyze Foxp3 expression. Numbers represent cells positively staining for the indicated surface markers. Similar results were obtained in three individual experiments. *B*, Nonadherent PBMCs from three healthy donors were stimulated with unirradiated DCs/allo-HCC in the absence or presence of HCCsp. Naive PBMCs from three healthy donors were also cultured in the absence or presence of HCCsp. CD4^+ ^T cells were gated to analyze CD25^high ^Foxp3^+ ^expression and the percentage of CD25^high^Foxp3^+ ^in CD4^+ ^population was shown. For each group, the mean ± SD is shown. *, Significant differences.

To investigate whether HCCsp-exposed fusion cells induce the generation of CD4^+ ^CD25^high ^Foxp3^+ ^Treg, nonadherent PBMCs from healthy donors were cocultured with unirradiated DCs/allo-HCC/sp at 10:1 ratio in the presence of HCCsp. Thereafter, the CD4^+ ^T cells were gated for analysis of CD25^+ ^population in CD4^+ ^T cells. Flow cytometry demonstrated that very high levels of CD25 expression were observed in CD4^+ ^T cells stimulated by unirradiated DCs/allo-HCC, as compared with those stimulated by unirradiated DCs/allo-HCC/sp. The low-affinity IL-2 receptor α-chain, CD25 is constitutively expressed on Treg and is also up-regulated on conventional antigen-activated T cells in the presence of IL-2, including the vaccine-induced antitumor effector T cells. Therefore, we examined the Foxp3 expression, a special marker for Treg [38] to confirm whether these up-regulated CD4^+ ^CD25^high ^T cells are Treg. As shown in Figure 4A, almost all of CD4^+ ^CD25^high ^T cells induced by unirradiated DCs/allo-HCC/sp expressed Foxp3 protein in the presence of HCCsp. Moreover, Foxp3 is also expressed in CD4^+^CD25^low/- ^T cells induced by unirradiated DCs/allo-HCC/sp. In contrast, there was about 50% reduction in Foxp3 expression among CD4^+ ^CD25^high ^T cells generated by unirradiated DCs/allo-HCC in the absence of HCCsp (Figure 4A). We also found that coculture of T cells with unirradiated DCs/allo-HCC/sp in the presence of HCCsp caused about 2-fold increase of CD25^high+ ^Foxp3^+^T cells among all CD4^+ ^T cells, as compared with those generated by unirradiated DCs/allo-HCC in the absence of HCCsp (Figure 4B). Taken together, these results suggest that DCs/allo-HCC/sp have the tendency to generate CD4^+ ^CD25^high ^Foxp3^+ ^T cells in the presence of the supernatants.

### Effect of autologous FCs vaccination on the phenotype of DCs

**Figure 5:**
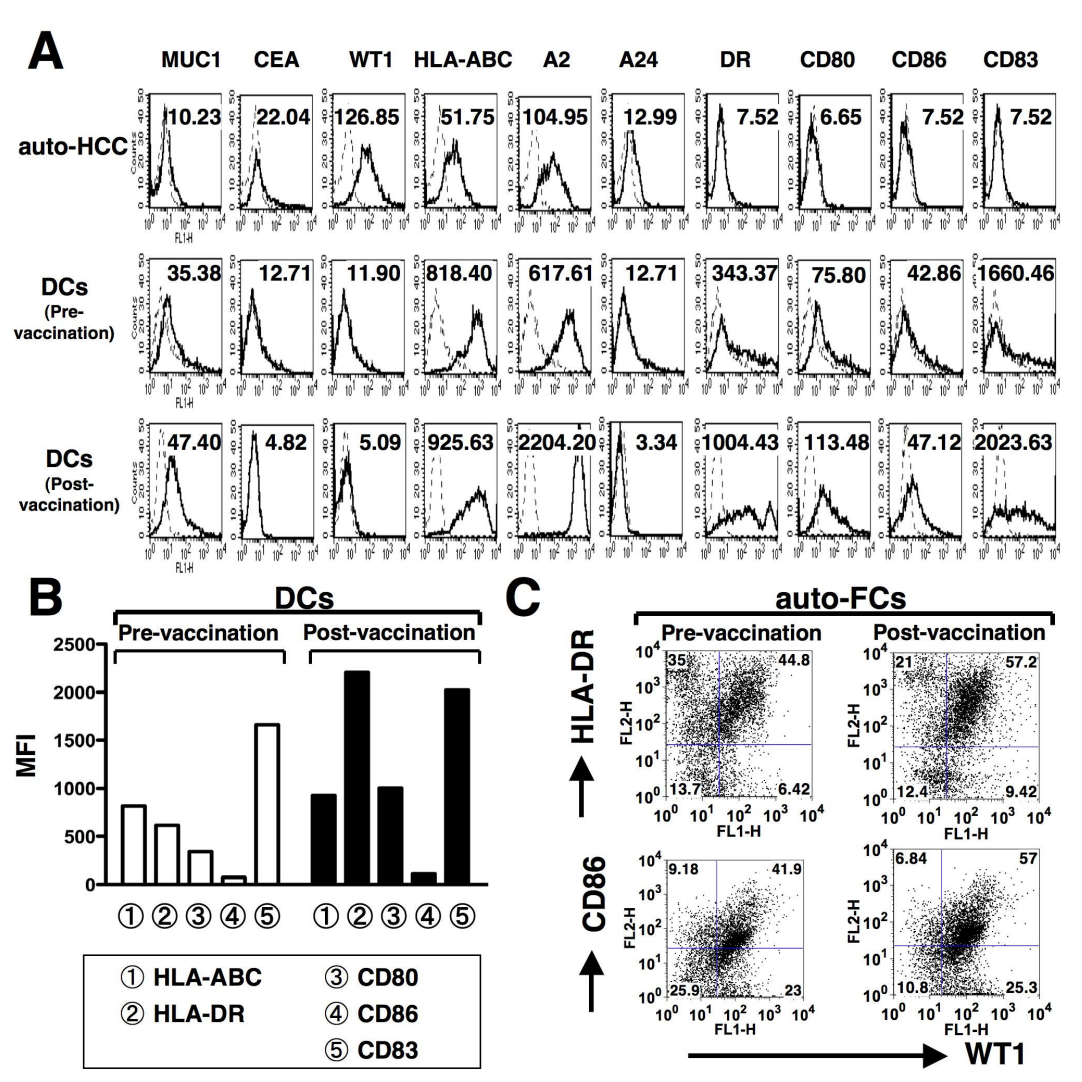
**Phenotypic characterization of DCs/auto-HCC. ***A*, Autologous HCC cells (auto-HCC) and DCs generated before and after vaccination were analyzed by flow cytometry for expression of the indicated antigens. Thin line, isotype control; thick line, indicated antigens. Numerical values show the MFIs of indicated antigens in DCs. *B*, MFIs of HLA-ABC, HLA-DR, CD80, CD86, and CD83 in DCs generated before and after vaccination were analyzed. *C*, Fusions of auto-HCC and DCs (before or after vaccination) were analyzed by 2-color flow cytometry for dual expression of WT1 and HLA-DR/CD86. Numbers represent cells positively staining for the indicated surface markers.

The HCC patient was vaccinated with autologous FCs nine times. Autologous HCC cells expressed high levels of WT1 and HLA-ABC (HLA-A2+/A24-) and low levels of CEA but not HLA-DR, costimulatory molecules (CD80 and CD86), and maturation marker, CD83 (Figure 5A). Before the vaccination and one month after the ninth vaccination, PBMCs were collected and frozen in liquid nitrogen until analysis. The phenotype of both DCs generated before and after vaccination was analyzed in the same set of experiments. After the ninth vaccination, the DCs displayed a characteristic phenotype with increased expression of HLA-DR, CD80, and CD83, as compared with that obtained before vaccination (Figure 5A and 5B). Before vaccination, 44.8 and 41.9% of autologous FCs were positive for WT1 and HLA-DR/CD86, respectively. After vaccination, however, the double-positive population was increased to 57.2 and 57.0%, respectively (Figure 5C).

### Immunological responses induced by autologous FCs vaccine

**Figure 6:**
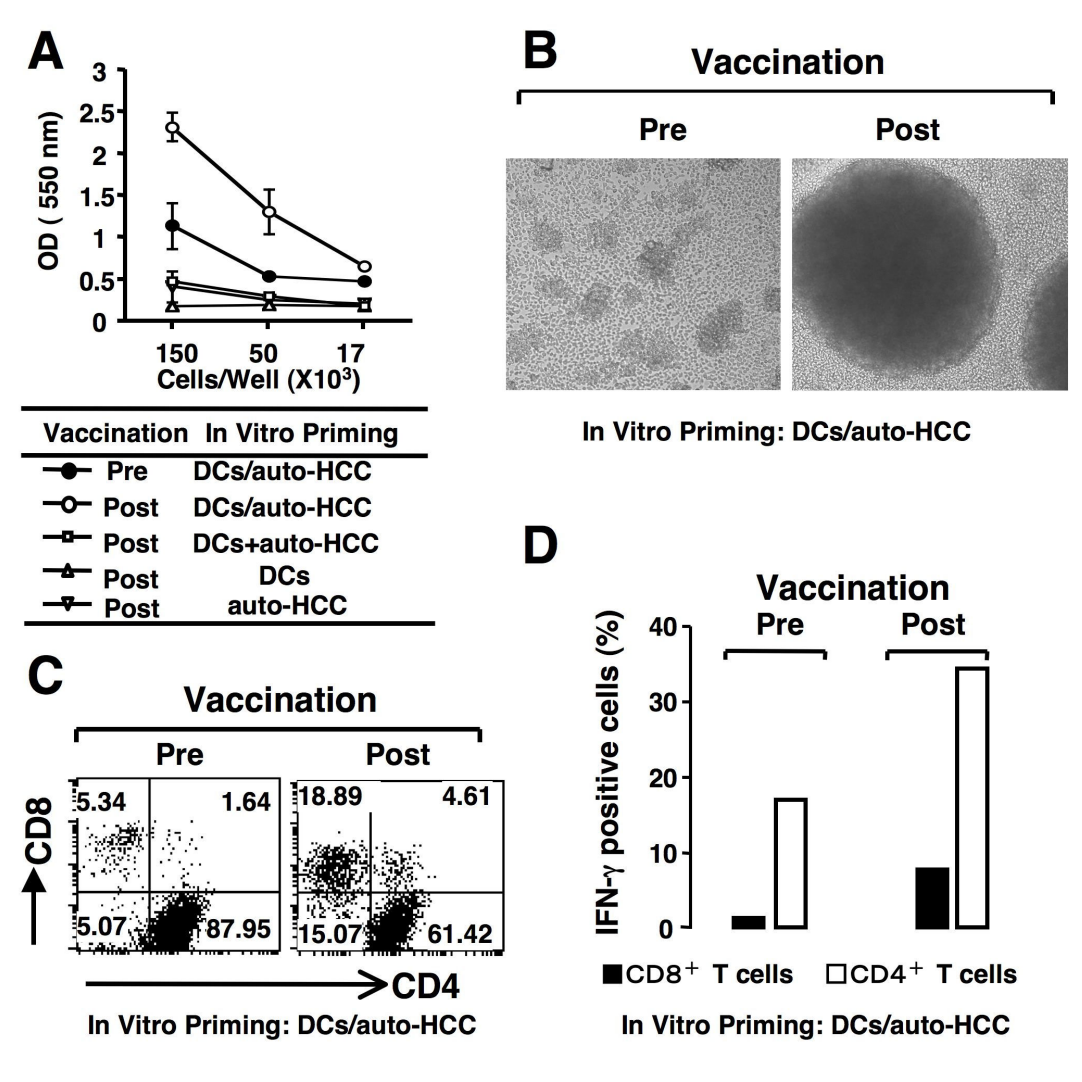
**The ability of vaccination with autologous FCs to stimulate T cells**. *A*, Nonadherent PBMCs obtained before and after vaccination were stimulated with unirradiated DCs/auto-HCC, DCs, auto-HCC cells, or an unfused mixture of both for 7 days. T-cell proliferation assay was performed by Cell Titer 96 Nonradioactive Cell Proliferation Assay Kit according to the protocol. *B*, Nonadherent PBMCs obtained before and after vaccination were stimulated with unirradiated DCs/auto-HCC and examined under a microscope (magnification, ×10). Similar results were obtained in three individual experiments. *C*, Nonadherent PBMCs obtained before (left panel) and after vaccination (right panel) were stimulated with unirradiated DCs/auto-HCC and stained with FITC-conjugated anti-CD4 and PE-conjugated anti-CD8 mAb. Numbers represent cells positively staining for the indicated surface markers. D, Nonadherent PBMCs obtained before (left panel) and after vaccination (right panel) were stimulated with unirradiated DCs/auto-HCC. Percentage of IFN-γ-positive CD4^+ ^or CD8^+ ^T cells was assessed. For each group, the mean ± SD of three experiments is shown.

The HCC patient was vaccinated nine times and immunological responses to the autologous vaccination were investigated. We first assessed the ability of autologous FCs vaccination to stimulate T cells. After the ninth vaccination, unirradiated DCs/auto-HCC stimulated T-cell proliferation responses more vigorously than did before vaccination. (Figure 6A). In addition, unirradiated DCs/auto-HCC stimulated larger cluster formations of T cells when compared with those obtained before vaccination (Figure 6B). Furthermore, coculture of T cells obtained after vaccination with DCs/auto-HCC resulted in an evolution of CD8^+ ^T cell populations from 5.34% to 18. 89% in vitro (Figure 6C). In contrast, autologous DCs or the HCC cells were not able to stimulate the T cells (Figure 6A).

We next examined the quality of CD4^+ ^and CD8^+ ^T cells from the HCC patient vaccinated by autologous FCs. When nonadherent PBMCs obtained before vaccination were restimulated with unirradiated DCs/auto-HCC in vitro, the expression of IFN-γ in both CD4^+ ^and CD8^+ ^T cells were much lower (Figure 6D). In contrast, the expression of IFN-γ in CD4^+ ^and CD8^+ ^T cells significantly increased after vaccination (Figure 6D). The low levels of IL-10 expression in T cells did not impair the production of IFN-γ (data not shown). These results suggest that vaccination with autologous FCs improves the immune responses in the patient.

### Induction of HCC cells-specific CTL by vaccination with autologous FCs

**Figure 7:**
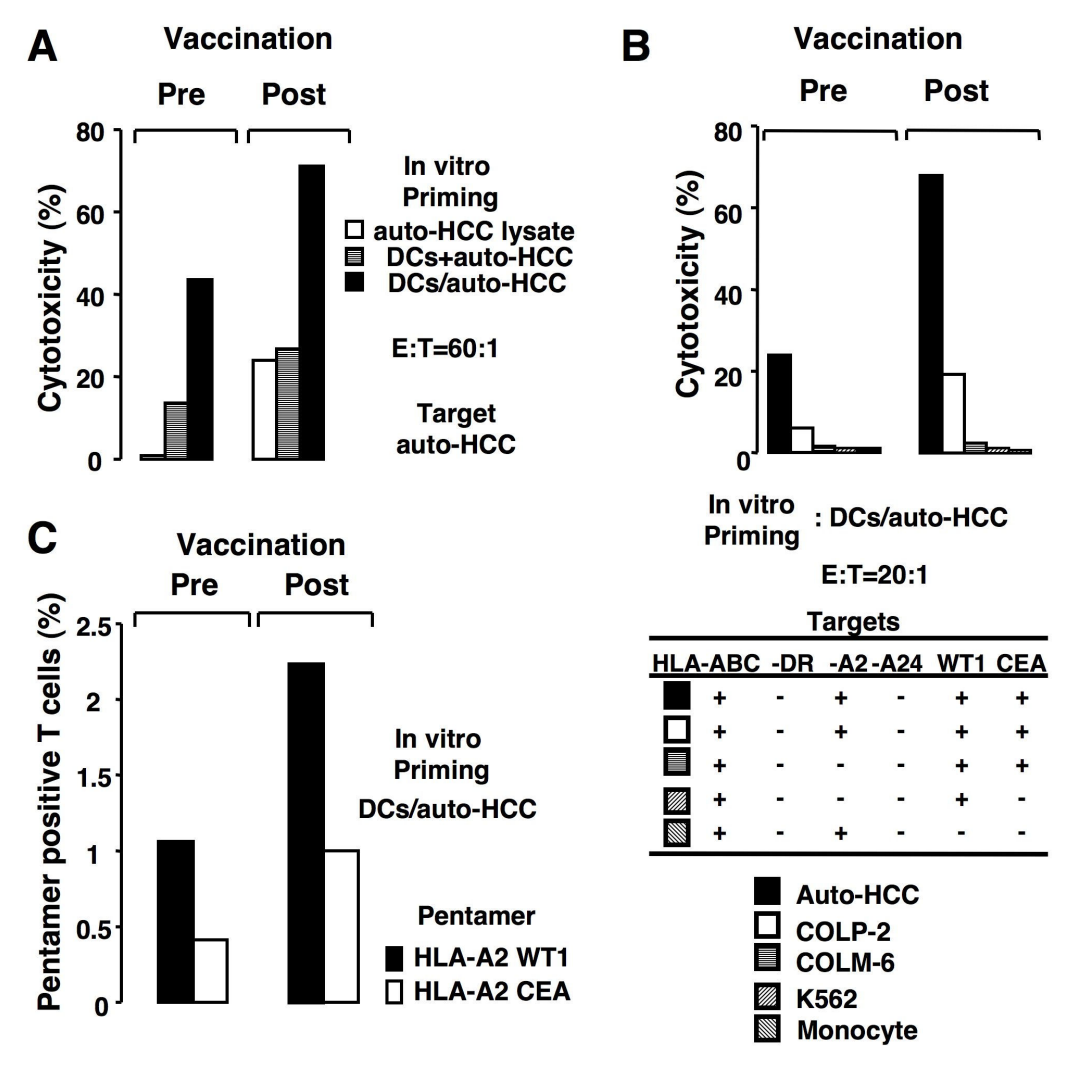
**Induction of CEA- and WT1-reactive T cells by vaccination with autologous FCs**. *A*, Nonadherent PBMCs obtained before (left panel) and after vaccination (right panel) were stimulated with auto-HCC cells lysates, DCs mixed with auto-HCC, or unirradiated DCs/auto-HCC. T cells were cocultured with the HCC cells at a ratio of 60: 1. *B*, Nonadherent PBMCs obtained before (left panel) and after vaccination (right panel) were stimulated by unirradiated DCs/auto-HCC. T cells were cocultured with the HCC cells, COLP-2, COLM-6, K562, or autologous monocytes at a ratio of 20:1. Percentage of cytotoxicity (mean ± SD of 3 replicates) was determined by flow cytometry-CTL assay. *C*, Nonadherent PBMCs (HLA-A2+/A24-) obtained before (left panel) and after vaccination (right pane) were stimulated with unirradiated DCs/auto-HCC. T cells were analyzed by HLA-A2/WT1 or HLA-A2/CEA pentameric assay. *, Significant differences.

We next examined whether fusion cell vaccination could augment the induction of HCC cells-specific CTL in the patient. Before vaccination, coculture of nonadherent PBMCs with unirradiated DCs/auto-HCC resulted in low levels of CTL induction against autologous HCC cells in vitro (Figure 7A). However, the CTL responses against autologous HCC cells were significantly augmented after vaccination (Figure 7A and 7B). Preincubation of the autologous HCC cells with anti-HLA-ABC mAb inhibited the lysis, suggesting the MHC class I restriction (data not shown). In contrast, there was minimal lysis of autologous HCC cells by nonadherent PBMCs obtained before vaccination cocultured with the HCC cells lysates, an unfused mixture of DCs and the HCC cells (Figure 7A), DCs alone, or the HCC cells alone (data not shown). Moreover, nonadherent PBMCs obtained after vaccination stimulated with the HCC cells lysates have considerable cytotoxic activity while no cytotoxicity is observed using those obtained before vaccination (Figure 7A). These results suggest that vaccination with autologous FCs has the potential to increase CTL precursors against autologous HCC cells in the patient.

To assess the antigen specificity and HLA restriction elements of CTL induced by vaccination with autologous FCs (HLA-A2+/A24-), we used CTL assay using autologous HCC cells and multiple allogeneic cell lines as targets. As shown in Figure 7B, T cells from after vaccination stimulated by unirradiated DCs/auto-HCC lysed not only the HCC cells (HLA-A2+/A24-, WT1+, and CEA+) but also HLA class I-semimatched colorectal carcinoma cell line, COLP-2 (HLA-A2+/A24-) endogenously expressing WT1 and CEA. By contrast, no lysis of allogeneic colorectal carcinoma cell line, COLM-6 (HLA-A2- and A24-, WT1+ and CEA+) was observed, suggesting that HLA-A2 was, at least in part, the restriction element of CTL (Figure 7B). In addition, CTL induced by unirradiated DCs/auto-HCC did not lyse autologous monocytes or NK-sensitive K562, indicating the selectivity for lysis of autologous HCC cells (Figure 7B). Furthermore, the pentameric assay confirmed that the population of WT1- and CEA-reactive CD8^+ ^T cells was augmented by the fusion cell vaccination. Before vaccination, coculture of nonadherent PBMCs with unirradiated DCs/auto-HCC resulted in 1.06% of WT1- and 0.41% of CEA- reactive CD8^+ ^T cells in HLA-A2 restrictive manner (Figure 7C). In contrast, about 2-fold increase in the percentage of WT1- and CEA-reactive CD8^+ ^T cells was observed after the ninth vaccination. No positive T cells were detected when an irrelevant pentamer was used or minimal antigen-positive T cells were detected when T cells were cocultured with DCs alone, the HCC cells alone, or an unfused mixture of DCs and the HCC cells in vitro (data not shown). Taken together, these results indicate that vaccination with autologous FCs is able to enhance the induction of WT1- and CEA-reactive CD8^+ ^T cells.

## Discussion

The present study provides first evidences that soluble factors derived from the HCC cells inhibit maturation of DCs and DCs/tumor fusion cells. These fusion cells, in turn, promote the induction of CD4^+ ^CD25^high ^Foxp3^+ ^Treg and impair induction of antigen-specific CTL. Although DCs from the patient with advanced HCC exhibit functional impairment, fusion cell vaccine improves DCs function and induces augmented antigen-specific polyclonal CTL.

Because the HCC patient had chronic active hepatitis based on carrier state of hepatitis B virus, the immune responses may be poorly reactive to autologous HCC cells. DCs from hepatitis B virus (HBV) carriers have been reported to exhibit functional impairment [39]. Possible explanations for this phenomenon are infection of HBV into DCs or alteration of DCs function by HBV and HCC itself [39]. This process is mostly related to HCC-derived soluble factors, several of which have been identified. Decreased function of DCs is one potential mechanism by which tumor evade the host's immune responses. Immature DCs are one of the mediators of tolerance induction. In peripheral lymphoid organs immature DCs are incapable of mobilizing CTL responses and have been reported to induce tolerance. In contrast, if a stimulus for DCs activation is sufficiently coadministered with antigens, mature DCs express high levels of costimulatory molecules, resulting in priming of antigen-specific CTL induction rather than Treg [25]. Therefore, we investigated whether supernatants derived from the HCC cells affect the function and maturation of DCs. The data show that exposure of immature DCs to the supernatants results in down-regulation of HLA-DR and costimulatory molecules (CD80), and maturation marker (CD83). The down-regulation of surface molecules on DCs by the supernatants may have negative effects in the interaction between T cells and DCs at the first phase of immunological synapse formation, hence decreasing the DC-dependent T-cell activation and proliferation in patients with advanced HCC. TLR agonists are potent activators of innate immune responses, inducing DCs maturation and inflammatory cytokine secretion by innate immune cells and as a consequence they promote antitumor immune responses when coadministered with antigens. It has been demonstrated that OK-432 promotes functional maturation of DCs through the TLR4 pathway to enhance antigen-specific CTL responses [25,40].

Lipopolysaccharide (LPS)-mediated TLR4 signaling also leads to maturation of DCs [25,40]. However, clinical use of LPS is limited due to potential toxicity. On the other hand, OK-432 is an agent of good manufacturing practice grade and has been widely used in patients with cancer [25,40]. Therefore, we have used OK-432 to stimulate immature DCs to determine whether the HCC cell culture supernatants have suppressive effects on DCs maturation. Immature DCs generated in the presence of the supernatants were unable to become fully mature after OK-432 stimulation, suggesting that administration of OK-432 alone cannot sufficiently help to induce DCs maturation in the presence of the immunosuppressive molecules produced by the HCC cells. This phenomenon is consistent with previous findings that tumor cells secrete many immunosuppressive cytokines and chemokines (IL-6, IL-10, and TGF-β) [41,42].

Moreover, tumor cells also secrete molecules such as AFP and MUC1, all of which affect the maturation and function of DCs [43,44]. In this study, the HCC cells used for fusion cell vaccination secrete low levels of TGF-β but no AFP, PIVKA-II, and MUC1 (data not shown). A recent study that has reported that DCs exposed with supernatants derived from HCC cell lines culture fail to undergo full maturation upon stimulation with LPS [45], also support our findings. Thus, if a stimulus for DCs activation is insufficiently administrated in the presence of the immunosuppressive molecules, DCs may fail to undergo full maturation, leading to induction of tolerance in patients with advanced HCC. Combined TLR agonists may be particularly essential for the full maturation of DCs in the local tumor microenvironment of cancer patients. In the present study, autologous fusion cells for vaccination were stimulated by TNF-α, but DCs used for the preclinical study were matured by OK-432. It has been reported that OK-432 promotes more functional maturation of DCs than that obtained with either LPS or a standard mixture of cytokines (TNF-α, IL-1β, IL-6, and PGE2) [25,46]. Therefore, even if we have used TNF-α in the preclinical study, similar results may be obtained in this experimental setting.

It has been reported that efficient CTL induction, which is particularly important in antitumor responses, required the stimulation of both CD4^+ ^and CD8^+ ^T cells [47]. Previously, we have reported that immature DCs fused with allogeneic tumor cell line present multiple TAAs and induce antitumor immunity against autologous tumor cells [33]. In this allogeneic human tumor cell line model, antigen-specific CTL responses induced by fusions of allogeneic tumor cell line and immature DCs have the same potency as those induced by fusions of autologous tumor cells and immature DCs in vitro [33]. Thus, to assess the functional capacity of fusion cells created from the HCC cells and DCs to stimulate CTL, we first fused the patient-derived HCC cells to immature DCs generated from healthy donors (DCs/allo-HCC) in the absence of the HCC cell culture supernatants. Donor's CD4^+ ^and CD8^+ ^T cells were strongly stimulated by DCs/allo-HCC with high levels of IFN-γ production, suggesting that antigens were presented through both MHC class I and class II pathways simultaneously. However, the INF-γ production and T-cell proliferation were abolished in CD4^+ ^and CD8^+ ^T cells primed by DCs/allo-HCC/sp in the presence of the supernatants. It could be argued whether the supernatants have a suppressive effect on DCs/allo-HCC/sp, on the stimulation of T cells by them, or an additive effect at both levels. Culture of naive PBMCs from healthy donors in the presence of the supernatants impaired T-cell proliferation (data not shown), suggesting that the supernatants have, at least in part, a suppressive effect on stimulation of T cells. Moreover, fusion cells created in the presence of the supernatants have an impaired characteristic phenotype and failed to undergo full activation upon stimulation with OK-432, suggesting that the supernatants also exhibit functional impairment of the fusion cells as APCs in the patient. OK-432 alone may be still insufficient to stimulate fusion cells in the local tumor microenvironment of the patient. In addition, DCs/allo-HCC/sp dual-expressed both WT1 and HLA-DR/CD86 at significantly lower levels than those obtained from DCs/allo-HCC, therefore, could not be optimal for CD4^+ ^and CD8^+ ^T cell stimulation in vitro. Because the levels of fusion efficiency are also closely correlated with antitumor immunity in a murine study (our unpublished data), the presence of the soluble factors may prevent the efficient induction of antitumor immunity. Recent studies that have demonstrated that HCC cell lines culture supernatants impaired therapeutic efficacy of the vaccine in tumor-bearing mice [45,48], support our findings that the supernatants impair the induction of CTL in vitro.

Important issues that must be addressed are how the HCC cell culture supernatants exert suppressive effects on CTL stimulation. There are increasing evidences that HCC cells-derived soluble factors promote the induction of tolerance through the generation of CD4^+ ^CD25^high^Foxp3^+ ^Treg subset, which is linked to compromised immune responses in patients with HCC [49,50]. Indeed, the potent immunosuppressive effects of Treg can explain the failure of many immunotherapeutic approaches to cancer [49]. Whether CD4^+ ^CD25^high ^Treg (naturally occurring Treg) are recruited from thymus and accumulated in HCC or whether HCC cells-derived soluble factors convert CD4^+ ^CD25^- ^T cells to CD4^+ ^CD25^high ^Treg in the periphery are currently unclear. In either way, HCC cells-derived soluble factors might play a central role in immune suppression mediated by Treg, suggesting that these factors interfere with DCs/tumor fusion approach and inhibit antitumor immune responses in patients with advanced HCC. Moreover, it has recently been reported that DCs are capable of inducing conversion of naive CD4^+ ^T cells to adaptive CD4^+ ^CD25^+ ^Foxp3^+ ^Treg in the presence of TGF-β [51]. The HCC cells used for fusions in the present study secrete low levels of TGF-β. Interestingly, coculture of nonadherent PBMCs from healthy donors with DCs/allo-HCC/sp in the presence of the supernatants resulted in generation of Treg. The abnormal function of DCs and differentiation into Treg in the local tumor microenvironment of the patient with advanced HCC may be due to the combined effects of numerous immunosuppressive cytokines and chemokines [45,48]. Although the effect of the HCC cell supernatants in the generation of Treg in vitro is demonstrated in the present study, little is known about the impact of fusion cell vaccination on generating Treg. The negative impact of fusion cell vaccine is still not clear in this experimental setting. A recent study has demonstrated that vaccination with DCs/tumor fusion cells producing TGF-β resulted in the induction of Treg in vivo and in vitro in a murine model [52].

Moreover, the blockade of TGF-β reduces Treg induction by the fusion cell vaccine and enhances antitumor immunity [52]. Depletion of human Treg before vaccination may also lead to enhanced antitumor immune responses in cancer patients [53]. If the immune suppressed environment in tumor is sufficiently improved, approaches for selective manipulation of the innate immune responses induced by combined TLR agonists may have more potential to promote DCs maturation and CTL over Treg generation [54]. Patients early in the course of the disease with low tumor burden and still an uncompromised immune system are expected to respond best to clinical responses by fusion cell vaccination.

Because DCs from the patient with advanced HCC exhibit functional impairment, the patient is poorly immune reactive to autologous tumor, as compared with healthy donors. Inhibition of DCs maturation could represent a frequent mechanism by which tumor cells will escape immune recognition. However, vaccination of the HCC patient with autologous fusion cells resulted in enhanced expression of HLA-DR, costimulatory molecules (CD80), and maturation marker (CD83) on DCs. The vaccination could recover functional impairment of DCs in the HCC patient. A recent study also has reported that the use of DCs-based cancer vaccines induces recovery of DCs function in metastatic cancer patients [55]. We also found before vaccination low levels of IFN-γ production in both CD4^+ ^and CD8^+ ^T cells, which were poorly reactive to autologous HCC cells. However, fusion cell vaccination elicited up-regulated production of IFN-γ in T cells. Importantly, coculture of nonadherent PBMCs obtained after vaccination with autologous fusion cells resulted in augmented CTL responses against autologous HCC cells, as compared with those obtained before vaccination. In addition, nonadherent PBMCs obtained after vaccination stimulated with even the HCC cells lysates have considerable levels of cytotoxic activity while no cytotoxicity is observed in those obtained before vaccination. Although the results from CTL assays are influenced by the in vitro stimulation procedures [56], it is reasonable to speculate that fusion cell vaccine can increase numbers of CTL precursor in the HCC patient. Interestingly, more than 2-fold increase of CTL responses specific for WT1 and CEA were observed after vaccination. Induction of antigen-specific polyclonal CTL is particularly important for eradicating tumor cells [24]. Thus, the multiple doses of vaccination may also have the potential to stimulate both CD4^+ ^and CD8^+ ^T cells and result in induction of antigen-specific polyclonal CTL responses in the patient.

Although hepatic lesions remained to be stable during vaccination, pulmonary metastases showed progression and died after seven month from the first vaccination. In spite of the immunological responses, defects of the clinical responses in the patient with advanced HCC may be caused by the immunosuppressive influences derived from tumor as shown in the present experimental setting. Even if HCC cells-specific CTL responses were observed in this study, CTL directed against the tumor might become functionally inactive when exposed to the local tumor microenvironment [39]. It has been shown that T cells in HCC lesions appear to contain Treg that accumulate locally and inhibit CTL responses [50]. Our in vitro results also support this notion and show that the HCC cell culture supernatants impair the DCs maturation, even if OK-432 is administrated, resulting the generation of Treg in vitro. The lack of therapeutic efficacy with fusion cell vaccine in the patient may be not due to low levels of CTL response but inhibitory activity by Treg. The HCC tissues are much more complex than the present experimental setting. Tumor tissues comprise not only of tumor cells but also of tumor-associated fibroblasts, vascular endothelial cells, extracellular matrix, and different variety of immune cells (DCs, macrophages, granulocytes, and NK cells), all of which are key regulators in tumorigenesis [57,58]. It has been demonstrated that tumor-associated fibroblasts and macrophages synthesize proteins, such as VEGF, TGF-β, and IL-10, all of which contribute to the local immunosuppressive environment [57-59]. Therefore, tumor rejection also can be achieved by modulation of tumor-stromal fibroblasts or by disturbance of the network [60,61]. Further studies are required to determine the inhibitory interactions among these cells and their secretary molecules on DCs differentiation and Treg generation.

The vaccine administrated to this HCC patient is fusions of autologous whole HCC cells and DCs; therefore, concern exists regarding the possible induction of hepatitis by this vaccination. However, no hepatitis was induced, as evidenced by the constant levels of serum AST and ALT. In the present study, vaccination of the HCC patient could be performed safely without significant adverse effects associated with the vaccination. To date, in reports on fusion cell vaccination, severe autoimmune diseases have not been induced by the treatment [28-32].

## Conclusion

Our results demonstrate that fusion cell vaccination can improve DCs function and induce CTL. However, supernatants derived from the HCC cells promoted the generation of Treg with enhanced immunosuppressive capacities in vitro. Treg may contribute to the attenuated CTL responses in the presence of the supernatants. A major obstacle to the development of any active immunotherapeutic approach to cancer is the immunosuppressive environment of the growing tumor. A combination of control of Treg and concomitant induction of CTL may be a more effective immunotherapy to reduce recurrence and prolong survival after surgery.

## Lists of Abbreviations

HCC: hepatocellular carcinoma; HCCsp: HCC cell culture supernatants; CEA: carcinoembryonic antigen; DC: dendritic cell; TAA: tumor-associated antigen; FC: fusion cell; DCs/auto-HCC: patient-derived DCs fused with autologous HCC cells; DCs/allo-HCC: healthy donor-derived DCs fused with allogeneic HCC cells; CTL: cytotoxic T lymphocytes; GM-CSF: granulocyte/macrophage colony-stimulating factor; mAb: monoclonal antibody; OD: optical density; PBMC: peripheral blood mononuclear cell; Treg: regulatory T cells

## Competing interests

The authors declare that they have no competing interests.

## Authors' contributions

SK, SH, and EH conceived of the study, participated in its design, coordination, and preparation of this manuscript. SK, MM, AT, EN, and, YN prepared reagents and PBMCs. SH, HK, and YS prepared fusion cells and performed vaccination of the patient. TO, KF, JG, and HT participated in its design and coordination. All authors have read and approved the final manuscript.
